# Identification and Characterisation of Canine Osteosarcoma Biomarkers and Therapeutic Targets

**DOI:** 10.3390/cancers18020262

**Published:** 2026-01-14

**Authors:** Jorja Jackson-Oxley, Aziza A. Alibhai, Rachel Thompson, Jennifer Lothion-Roy, Simone de Brot, Mark D. Dunning, Jennie N. Jeyapalan, Nigel P. Mongan, Catrin S. Rutland

**Affiliations:** 1 School of Veterinary Medicine and Sciences, Faculty of Medicine and Health Sciences, University of Nottingham, Nottingham LE12 5RD, UKplzjnj@exmail.nottingham.ac.uk (J.N.J.); 2 Biodiscovery Institute, Faculty of Medicine and Health Sciences, University of Nottingham, Nottingham NG7 2RD, UK; 3 COMPATH, Institute of Animal Pathology, University of Bern, CH-3012 Bern, Switzerland; 4 Willows Veterinary Centre and Referral Service, Solihull B90 4NH, UK; 5 Department of Pharmacology, Weill Cornell Medicine, New York, NY 10065, USA

**Keywords:** forkhead Box F1 (FOXF1), G protein-coupled receptor 64 (GPR64), matrix Metallopeptidase 12 (MMP-12), TOX high mobility group Box family member 3 (TOX3)

## Abstract

**Background:** Osteosarcoma (OSA) is the most common type of bone cancer in canines. Novel therapies are required to prevent the growth, survival, and metastatic progression of this cancer, to increase life expectancy of patients. Immunohistochemical (IHC) studies and RNA sequencing help us gain a deeper understanding into the molecular mechanisms of the disease. **Methods:** We previously compared canine OSA tissues with patient matched non-tumour tissues, revealing 442 overexpressed genes within the samples. The present research used IHC staining for four of these genes in OSA tissues: G protein-coupled receptor 64 (*GPR64*), TOX High Mobility Group Box Family Member 3 (*TOX3*), Matrix Metallopeptidase 12 (*MMP-12*), and Forkhead Box F1 (*FOXF1*). H-scoring was performed to quantitatively assess protein expression and qualitatively contextualise staining locations. Additional analyses addressed whether gender or anatomical location of lesions (axial or appendicular tumours) affected protein expression. cBioPortal was employed to analyse expression and genetic alterations in patients. **Results:** *GPR64*, *TOX3*, *MMP-12*, and *FOXF1* showed high mRNA expression and genetic alterations in people with OSA. GPR64, TOX3, MMP-12, and FOXF1 were all expressed in canine OSA with novel findings regarding cellular expression. Additionally, differential sex expression was revealed for GPR64 and TOX3. Potential biomarkers or therapeutic targets were identified. **Conclusions:** These studies, and subsequent analysis, have provided insights into the molecular mechanisms associated with OSA progression and revealed potential biomarkers for diagnostic and prognostic purposes. A deeper understanding of genetic and protein interactions will support and progress novel pathways towards diagnostic, prognostic, and treatment interventions for OSA in both veterinary and human medicine.

## Simple Summary

Osteosarcoma is a bone cancer that is commonly observed in dogs and also affects people. The cancer often spreads, metastasizes, before the original cancer is even diagnosed, making treatment more difficult, less efficient, and resulting in more patient deaths. Current therapeutic protocols, in both dogs and people, produce low survival rates, and hence new therapeutic regimens are required. Studying tumour tissue is the ‘gold standard’ technique for diagnosing many cancer types. In medical research, techniques including immunohistochemistry, which studies patient tumour specimens, help us understand how much protein cancer expresses and show its location within the tumour. The proteins investigated are termed ‘biomarkers’ if they help detect and monitor the disease, and can indicate whether treatments work or enable us to understand the disease better. The use of these techniques in veterinary medicine has not been implemented for osteosarcoma, as biomarkers have not yet been identified. This study, therefore, investigated the proteins from genes that were overexpressed in canine osteosarcoma tissue. This has helped us to understand bone cancer in dogs better and identify potential drug targets and diagnostic biomarkers.

## 1. Introduction

Osteosarcoma (OSA) is the most prevalent form of bone malignancy in dogs and has an incidence rate estimated at 13.9/100,000 population [1]. A bimodal age distribution is observed in the dogs affected, although dogs over 7 years old account for the majority of cases (80%) [2]. Lesion formation is more predominant within the male population and in neutered dogs [3,4]. Large and giant breeds (25–45 kg and >45 kg, respectively), such as Scottish Deerhounds, Leonbergers, Great Danes, and Rottweilers, are most at risk of lesion development, which has been attributed to their increased body mass and longer limbs [3,5,6,7]. Chondrodystrophic (short-legged) breeds were shown to have a lower incidence than non-chondrodystrophic breeds (long-legged) [3]. The most robust marker for dogs with chondrodystrophy is the presence of the autosomal dominant fibroblast 4 (*FGF4*) mutation on chromosome 12 (*FGF4L2*) [8]. Dogs with this mutation exhibit the phenotype of very short, long bones and intervertebral disc disease [9]. *FGF4L2* is displayed at a high frequency in chondrodystrophic, spaniel, and dachshund breeds, and it has been hypothesised that this protects these dogs from OSA development [3]. OSA has shown associations with enhanced oncogenic susceptibility during bone growth, identified via the occurrence of tumours within metaphyseal regions, highly proliferative areas, of weight-bearing bones (distal radius and proximal humerus) [10]. Skull length is also a predisposing factor for OSA [11]. Dolichocephalic (long skull length) breeds showed enhanced susceptibility to tumour formation (Odds ratio (OR) 2.72) compared to mesocephalic (average skull length) breeds, whereas brachycephalic breeds had reduced odds (OR 0.50) [11]. The *FGF4* mutation associated with chondrodystrophy in dogs also reduces neurocranium size [12]. Furthermore, brachycephaly in dogs has associations with the suppression of the BMP signalling pathway, due to a BMP3 missense mutation and a transposable element insertion in the SPARC-related modular calcium binding protein (SMOC) 2 gene [3]. BMP and FGF signalling tend to promote OSA development [3]. Reduced activity of these signalling pathways in brachycephalic and chondrodystrophic breeds is suggestive of a decrease in OSA occurrence [3].

Due to the diseases’ highly malignant tendencies, the prognosis for dogs with OSA is poor [13]. Once OSA has metastasised to secondary sites, dogs have an approximate survival rate of 20%, and patients have a median survival time of 3 to 4 months if treated by amputation or lesion excision alone [14,15]. OSA pathogenesis is difficult to distinguish as there are differing cellular origins, precursor lesions are frequently absent, and the tumours are genetically complex [16,17]. Clinical presentation of the disease in canines is predominantly variable degrees of lameness and swelling at the primary site [18]. Radiographs can be performed to facilitate a diagnosis, with characteristic features such as lytic and blastic bone lesions displaying a ‘sunburst effect’, and ‘Codman’s triangle’ due to periosteal elevation frequently identified [19]. Biopsies help provide not only diagnostic information but also tumour grading [19]. Both canine and human OSA are usually treated as standard via amputation or limb-sparing surgery, often with adjunct chemotherapy [14,20]. Immunotherapy may be offered where standard treatments have failed, especially in clinical trials, and a canine OSA USDA (Product Code 95A7.50) approved personalised T-cell therapy became available in 2025 [21]. Although amputation is more common, preservation of limb function via limb salvage surgery is frequently preferred in certain anatomical sites, especially for those with concurrent neurological or orthopaedic issues, as better return-to-function may be achieved [22]. In dogs, cisplatin or carboplatin has been used as a stand-alone therapy or alongside doxorubicin [14,23,24]. When administered post-amputation, cisplatin has shown a median survival rate of 9–11 months, with approximately 75–80% of patients developing fatal metastases by 2 years [15].

Immunohistochemistry (IHC) to characterise protein expression is a standardised diagnostic technique in human medicine, but is rarely used within a veterinary context. IHC can help aid tumour diagnosis, including grade, malignancy, identification of the lesions’ cellular lineage, and primary lesion location(s) prior to metastatic dissemination [25]. This technique is valuable for testing drug efficacy and detecting up- or downregulation of therapeutic targets [25]. A wide range of confirmed tumour markers, such as enzymes, tumour-specific antigens, oncogenes, tumour suppressor genes, and proliferative markers, in human medicine allows for this technique to be widely used across many tissue types [25]. Following IHC, histochemical scoring (H-scoring) is frequently conducted in many cancer types to semi-quantitatively assess protein expression of markers. H-scores are widely used in human medicine and provide vital insights when undertaking diagnostic and prognostic judgments, and they are also vital for decision-making concerning consequent therapeutic protocols [26]. For example, higher H-scores of epidermal growth factor receptor (EGFR) are often associated with shorter overall survival rates in patients with non-small cell lung cancer [27]. Likewise, breast cancer patients are often put into different treatment groups based on the H-score expression of various markers (including oestrogen receptor (ER), progesterone receptor (PR), and human epidermal growth factor receptor 2 (HER2)) [28]. IHC and H-scoring are beneficial tools for protein detection as opposed to other methods, such as Western blots, as protein expression can be measured in different subcellular locations (nuclear or cytoplasmic, as well as the stroma and membranes) [26].

Extensive research into both IHC and H-scoring must be performed prior to standardising IHC as a diagnostic tool in practice. In the veterinary field, research to identify markers is still in progress. Nevertheless, IHC applications are just as beneficial to this industry as they are in human medicine, although their implementation into practice will not be instantaneous once markers have been identified. This method is not just advantageous for canine OSA but also for other cancer types in various species, although antibody specificity may potentially pose a problem in some species and for specific markers.

The present study expands on previous work conducted by our research group by identifying and characterising expression of biomarkers that have the potential to be therapeutic targets and/or prognostic factors in canine OSA. *GPR64*, *TOX3*, *MMP-12*, and *FOXF1* RNA expression were previously shown to be expressed at significantly higher levels in canine OSA tissues compared to matched non-tumour bone [29]. These are of interest due to their associations with either OSA and/or other cancers in people, and because some already have potential therapeutic agents which could be developed for use in OSA. GPR64 is a member of the adhesion G protein-coupled receptor family (aGPCRs). Mutations in human aGPCRs have been evidenced within several cancer types, including OSA and bone cancer cell lines, but also in various bone disorders [30,31,32]. It is also notable that overexpression of GPR110 and GPR56 correlates with poorer survival rates in people with OSA [33,34]. TOX3 has been identified as a transcriptional factor of the protein transporter ATP Binding Cassette Subfamily G Member 2 (ABCG2), which has enhanced ABCG2 expression in colorectal cancer [35]. ABCG2 has been shown to be expressed highly in human OSA, contributing to significantly shorter overall patient survival times compared with normal ABCG2 expression levels [36]. A number of drugs indirectly inhibit TOX3, making it a valuable potential target in cancer therapy [37,38]. *MMP-12* overexpression has been human lung adenocarcinoma (LUAD), colorectal cancer, hepatocellular carcinoma (HCC), and OSA [39,40,41,42,43]. Potential therapeutic agents include all-trans retinoic acid (ATRA), which can reduce MMP-12 secretion, suppressing OSA cell migration [43], Aderamastat (FP-025), a selective MMP-12 inhibitor, and AZD1236, a dual MMP-9/MMP-12 inhibitor, which have not yet been used in cancer [44,45,46]. High expression levels of *FOXF1* have been observed in human OSA cell lines and tumours and are associated with lung metastasis [47]. It has also shown both anti- and protumour effects, especially in breast cancer in people [48,49]. This research all indicates that GPR64, TOX3, MMP-12, and FOXF1 are highly relevant as potential biomarkers and targets in canine and human OSA, and may also be important in other cancer types given their known expression to date. The aims of the present study were to determine GPR64, TOX3, MMP-12, and FOXF1 expression patterns utilising immunohistochemistry and H-scoring in OSA tissues from OSA canine patients. Additionally, analysis was conducted to determine whether differences in sex or anatomical location of the tumour affected protein expression. cBioPortal analysis was also conducted to determine whether genetic alterations were present in human patients with OSA.

## 2. Materials and Methods

### 2.1. Specimen Preparation and Ethics

Canine OSA tissue analysis underwent approval from the University of Nottingham School of Veterinary Medicine and Science ethics committee (ethics permission numbers 1823 160714, 959 130925, 3832 230502). International and national ethical standards were adhered to. OSA samples were excised from Rottweilers euthanised for reasons unrelated to this present study. A definitive OSA diagnosis was provided by a board-certified histopathologist.

All *n* = 25 OSA samples (described in Table S1) were acquired in the form of formalin-fixed, paraffin-embedded (FFPE) tissue blocks; samples were not decalcified, and full-face sections were utilised. The male population (*n* = 9/25) within this study ages ranged from 4.5 to 9 years (6.72 ± 0.4818; Figure S1A), with three animals neutered, one remaining entire, and the rest having an unspecified neutering status. The female population (*n* = 15/25) ages ranged between 4.83 and 12 years (9.06 ± 0.5639; Figure S1A), of which twelve were neutered, one remained entire, and the rest did not have a confirmed neutering status. One sample (*n* = 1/25) did not have a confirmed sex. The majority of samples were located within the appendicular skeleton (*n* = 19/25), with the minority of samples being collected from axial locations (*n* = 6/25). The ages and sexes of the samples were tested for normality to confirm whether the data were normally distributed; Shapiro–Wilk: *W* = 0.9807, *p* = 0.9662 (ns; male), *W* = 0.9578, *p* = 0.6867 (ns; female). A statistical *t*-test was conducted to check for differences between the ages of male and female samples, which concluded the female ages were significantly higher than male ages in this study (*t*-test: *p* = 0.01909, Supplementary Figure S1A).

### 2.2. Immunohistochemistry and Microscopy

*GPR64*, *TOX3*, *MMP-12*, and *FOXF1* were identified as significantly overexpressed genes in canine OSA compared to non-tumour patient-matched tissue samples following RNA sequencing analysis conducted by Simpson et al. [29]. Hence, IHC and H-scoring techniques were conducted to investigate subcellular expressions of their corresponding proteins. Antibodies were selected using BLAST (v2.16.0), a gene sequencing alignment software. The antibodies for GPR64 [50], TOX3 [51], MMP-12 [52], and FOXF1 [53] were unconjugated rabbit polyclonal antibodies with proven IHC applications. The BLAST results were: GPR64 100% coverage, 81.25% identity, TOX3 100% coverage, 95.95% identity; MMP-12 100% coverage, 76.74% identity; and FOXF1 100% coverage, 94% identity.

IHC was performed using the Leica Novolink Polymer Detection Kit (Leica, Wetzlar, Germany) in concordance with the manufacturer’s protocol using Rottweiler OSA FFPE blocks sectioned at 7 µm thickness using an N35 microtome blade (FEATHER Safety Razor Co. Ltd., Osaka, Japan). Dilution optimisations resulted in final dilutions of 1:100 for GPR64 (PA565594; Invitrogen, Cambridge, UK), TOX3 (PA559151; Invitrogen, UK), and FOXF1 (PA5-40516; Invitrogen, UK), and a 1 in 200 dilution for MMP-12 (22989-1-AP; Proteintech, Manchester, UK). Five percent foetal calf serum was used to dilute primary antibodies. Negative control samples were incubated in foetal calf serum only, and no primary antibody was used. Systematic random sampling techniques were used when conducting microscopy (Leica, Wetzlar, Germany) at 400× magnification, *n* = 5 photomicrographs per section. These underwent H-scoring to allow for semi-quantitative analysis of protein expression within nuclear and cytoplasmic subcellular locations in canine OSA tissues. Qualitative descriptions of staining patterns and identification of structures were also conducted using photomicrographs at 400× and 50× magnification.

### 2.3. H-Scoring, Statistical and Bioinformatic Analysis

H-scoring is deemed the ‘gold standard’ method of immunohistochemical interpretation within human medicine, but it is not commonly used within the veterinary industry yet. Protein expression is semi-quantitatively assessed based on the intensity and coverage of staining present within tissues. Scores, 0, 1+, 2+, or 3+ (representing absent, weak, moderate, or strong staining), were manually assessed for nuclear and cytoplasmic staining independently. H-scores of between 0 and 300 were calculated using the following formula: H-score = [1 × (% cells 1+) + 2 × (% cells 2+) + 3 × (% cells 3+)]. H-scoring was undertaken by one researcher for each of the proteins of interest (GPR64, TOX3, MMP-12, and FOXF1). A second scorer randomly scored 10% of the samples to ensure concordance. An intraclass correlation coefficient (ICC) of >90% was achieved for all proteins, confirming interpretation consistency.

**Figure 1:**
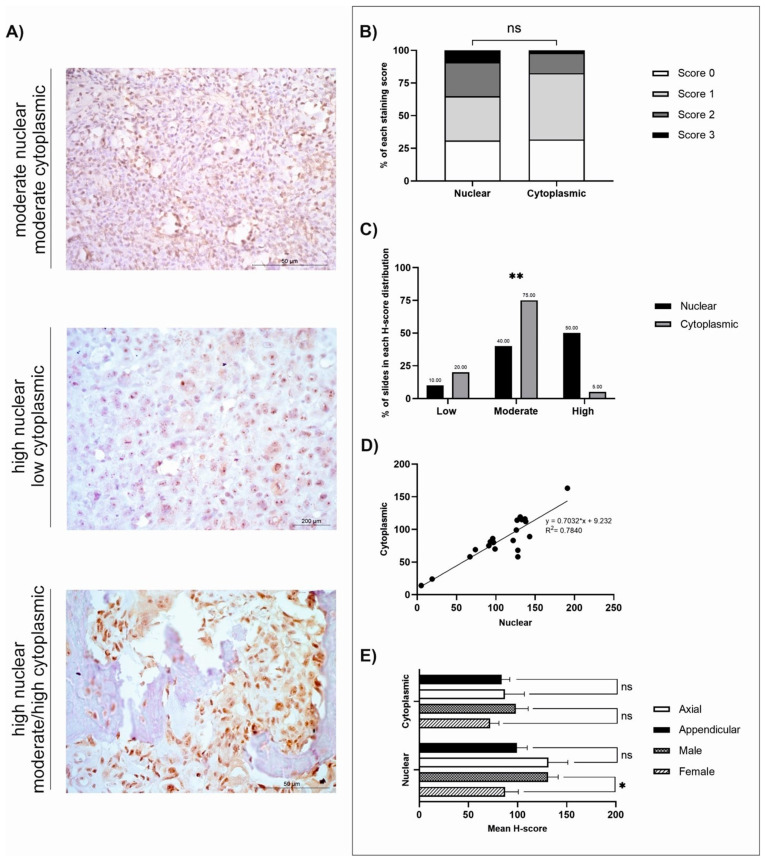
GPR64 H-score analysis (*n* = 20). (**A**) Photomicrographs of GPR64 immunohistochemistry at ×400 magnification showing varying intensities of positive protein expression. Scale bars represent 50 µm. (**B**) Average nuclear and cytoplasmic H-scores (scored 0–3); *t*-test: *p* > 0.05. (**C**) Nuclear and cytoplasmic H-score distributions (groups based on 1/3 of the largest range of scores): low (≤62), moderate (63–124), high (≥125); Chi-squared: *p* < 0.0062. Post hoc test using Fisher’s exact approach, Bonferroni-corrected significance level (*p* = 0.008333), low-low (*p* = 0.6614), moderate-moderate (*p* < 0.0.056), high-high (*p* < 0.0033). (**D**) Correlation between the nuclear and cytoplasmic H-scores. (**E**) GPR64 immunostaining nuclear and cytoplasmic H-score, gender, and anatomical location. GPR64 positive nuclear and cytoplasmic H-scores of tissues from male (*n* = 8) versus female (*n* = 11) dogs, as well as neoplasms excised from axial (*n* = 5) compared to appendicular (*n* = 15) locations. *p* ≥ 0.05 (ns), *p* < 0.05 (*), *p* < 0.01 (**).

**Figure 2:**
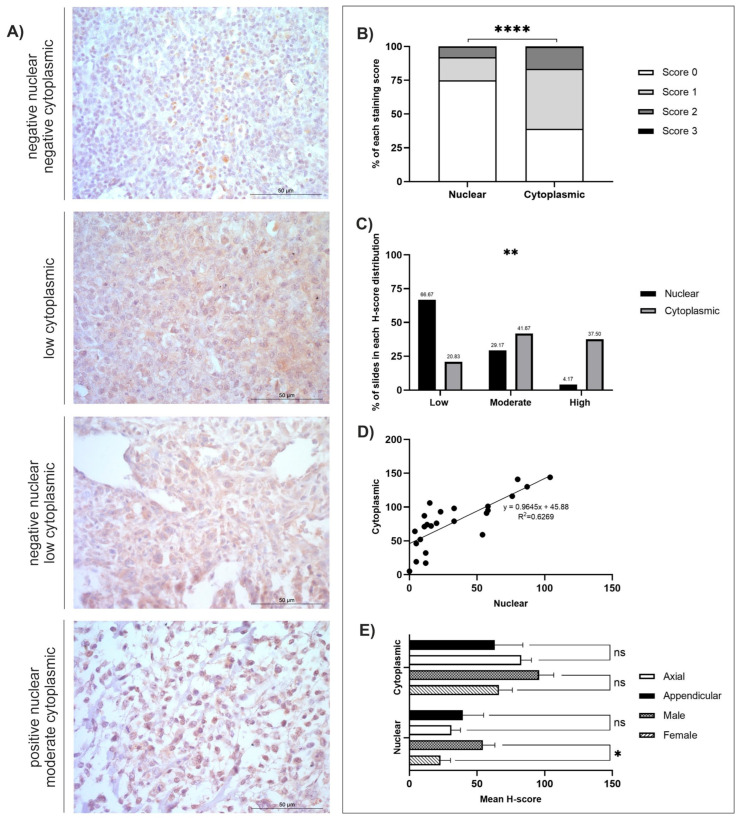
TOX3 H-score analysis (*n* = 24). (**A**) Photomicrographs of TOX3 immunohistochemistry, ×400 magnification, showing varying intensities of positive protein expression. Scale bars represent 50 µm. (**B**) Average nuclear and cytoplasmic H-scores (scored 0–3); *t*-test: *p* < 0.0001. (**C**) Distribution of nuclear and cytoplasmic H-scores (groups represented 1/3 of the largest range of scores); low (≤46), moderate (47–92), high (≥93); Chi-squared: *p* < 0.0018. Post hoc test using Fisher’s exact approach, Bonferroni-corrected significance level (*p* = 0.008333), low-low (*p* < 0.0032), moderate-moderate (*p* = 0.5469), high-high (*p* < 0.0102). (**D**) Correlation between the nuclear and cytoplasmic H-scores. (**E**) TOX3 immunostaining nuclear and cytoplasmic H-score, gender, and anatomical location. TOX3 positive nuclear and cytoplasmic H-scores of tissues from male (*n* = 8) compared to female (*n* = 15) dogs as well as neoplasms excised from axial (*n* = 6) compared to appendicular (*n* = 18) locations. *p* ≥ 0.05 (ns), *p* < 0.05 (*), *p* < 0.01 (**). *p* < 0.0001 (****).

**Figure 3:**
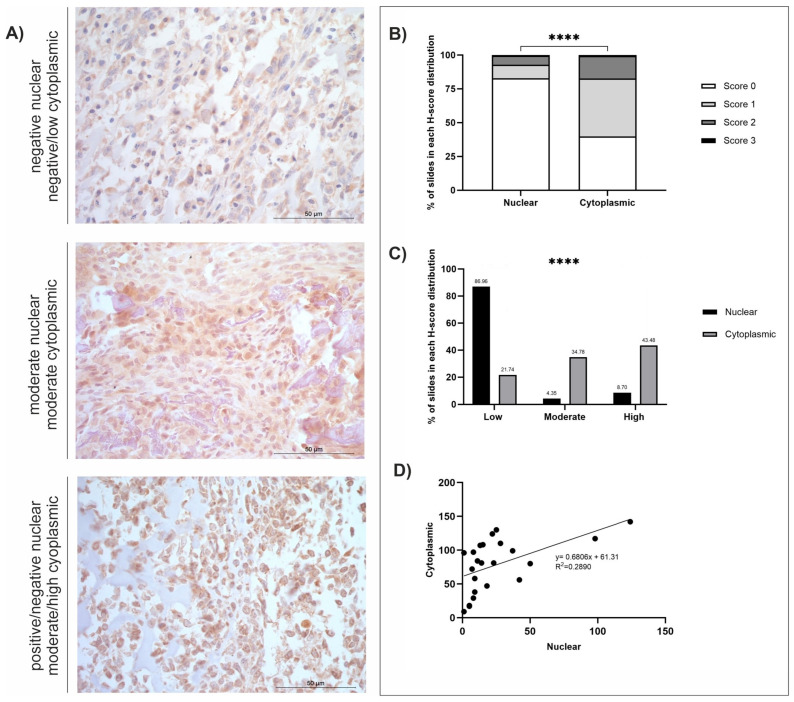
MMP-12 H-score analysis (*n* = 23). (**A**) Photomicrographs of MMP12 immunohistochemistry, ×400 magnification, showing varying intensities of positive protein expression. Scale bars represent 50 µm. (**B**) Average nuclear and cytoplasmic H-scores (scored 0–3); *t*-test: *p* < 0.0001. (**C**) Distribution of nuclear and cytoplasmic H-scores (groups represented by 1/3 of the largest range of scores): low (≤44), moderate (45–88), high (≥89); Chi-squared: *p* < 0.0001. Post hoc test using Fisher’s exact approach, Bonferroni-corrected significance level (*p* = 0.008333), low-low (*p* < 0.0001), moderate-moderate (*p* < 0.02), high-high (*p* < 0.0165). (**D**) Correlation between nuclear and cytoplasmic H-scores. *p* < 0.0001 (****).

**Figure 4:**
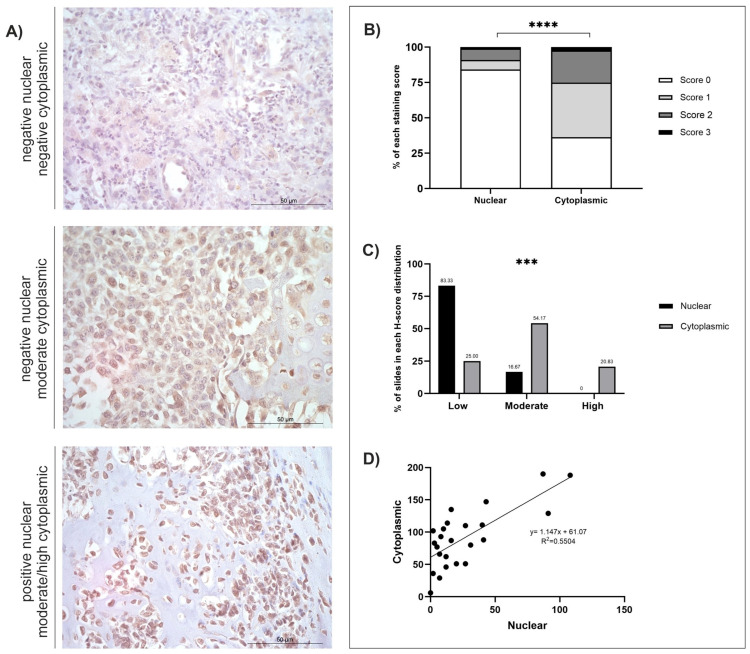
FOXF1 H-score analysis (*n* = 24). (**A**) Photomicrographs of FOXF1 immunohistochemistry, ×400 magnification, showing varying intensities of positive protein expression. Scale bars represent 50 µm. (**B**) Average nuclear and cytoplasmic H-scores (scored 0–3); *t*-test: *p* < 0.0001. (**C**) Distribution of nuclear and cytoplasmic H-scores (groups calculated by performing a 1/3 of the largest range of scores): low (≤61), moderate (62–122), high (≥123); Chi-squared: *p* < 0.0002. Post hoc test using Fisher’s exact approach, Bonferroni-corrected significance level (*p* = 0.008333), low-low (*p* < 0.0001), moderate-moderate (*p* < 0.0001), high-high (*p* < 0.0001). (**D**) Correlation between nuclear and cytoplasmic H-scores. *p* < 0.001 (***), *p* < 0.0001 (****).

The mean, standard error of the mean (SEM), and range (minimum-maximum scores) were calculated (Table 1). Staining distribution patterns, diffuse, multifocal, or focal, were also assigned for each sample for each antibody (Table 1). Both nuclear and cytoplasmic subcellular locations were separately assigned a predominant staining intensity (absent, low, moderate, or high) following H-score classifications (Table 2). The low/moderate/high classifications were assessed using the greatest range (either nuclear or cytoplasmic) for each antibody: GPR64 = low (≤62), moderate (63–124), high (≥125); TOX3 = low (≤46), moderate (47–92), high (≥93); MMP-12 = low (≤44), moderate (45–88), high (≥89); FOXF1 = low (≤61), moderate (62–122), high (≥123). Individual graphs to demonstrate staining distributions and H-scores were generated for GPR64, TOX3, MMP-12, and FOXF1 (Figure 1, Figure 2, Figure 3 and Figure 4). GraphPad Prism (GraphPad Software, version 9.4.0, San Diego, CA, USA) was used to calculate statistical *t*-tests to compare nuclear and cytoplasmic H-score differences, subcellular female versus male H-scores, and anatomical location (axial versus appendicular) nuclear and cytoplasmic H-scores. In addition, chi-squared tests, alongside post hoc analysis [54], were performed to identify differences between the low/moderate/high groups. Qualitative descriptions to contextualise immunohistochemical staining patterns were also recorded.

cBioPortal [55] was used to identify genetic alterations of *GPR64*, *TOX3*, *MMP*-*12*, and *FOXF1*, including mutations, putative copy-number alterations (from GISTIC), and mRNA expression (transcripts per million (TPM) z-scores with a threshold of ±2.0) within human OSA tissues based on the TARGET GDC 2025 cohort (*n* = 153 patients and *n* = 159 samples).

## 3. Results

### 3.1. Analysis of Protein Levels of Four Genes Highly Expressed in Canine Osteosarcomas

*GPR64*, *TOX3*, *MMP-12*, and *FOXF1* were all significantly overexpressed at the mRNA level in canine OSA tumours compared to patient-matched, non-tumour tissues (*p* = 0.0009, *p* = 0.0002, *p* = 0.0001, and *p* = 0.0005, respectively, and log2 Fold Change 3.669, 3.779, 3.785, and 3.894, respectively) [29]. Summaries of the IHC staining results of the four respective proteins of interest are displayed in Table 1 and Table 2. All four antibodies displayed diffuse staining across tissues, and observable multifocal staining was also evident in samples stained with FOXF1. In-depth analyses were conducted for GPR64, TOX3, MMP-12, and FOXF1, as shown in Figure 1, Figure 2, Figure 3 and Figure 4 and Supplementary Figure S1, depicting score distributions, nuclear and cytoplasmic correlations, and comparisons between sexes and anatomical locations of the lesions in subcellular staining.

### 3.2. GPR64

GPR64 staining was diffuse in canine OSA, and there was no significant difference when investigating nuclear and cytoplasmic H-score totals (*t*-test, *p* = 0.0726; Figure 1B). Once grouped into low, moderate, and high classifications, there was a significant difference between classifications (Χ^2^ = 50.80, df = 2, *p* < 0.0062). Post hoc analysis using the Fisher exact approach revealed a significant difference between nuclear and cytoplasmic high scores (*p* < 0.0033) with a Bonferroni-corrected significance level of *p* = 0.008333 (Figure 1C) [54]. All tissues (*n* = 20) displayed positive subcellular staining of GPR64 (both nuclear and cytoplasmic) at varying intensities. The majority of samples displayed moderate cytoplasmic staining (15/20; 75%), and 4/20 (20%) displayed low cytoplasmic staining (Figure 1C). Additionally, 1/20 tissues (5%) displayed both high nuclear and high cytoplasmic staining. Nuclear staining was high in half of the tissues (10/20; 50%), moderate in 8/20 (40%), and low in 2/20 (10%) (Figure 1C). There was a strong positive correlation between nuclear and cytoplasmic GPR64 expression (R^2^ = 0.784; Figure 1D). Statistical *t*-tests comparing mean nuclear and cytoplasmic H-scores between axial and appendicular locations, as well as between sexes, were conducted. Results revealed significantly higher nuclear GPR64 expression in males than females (*t*-test, 0.0267; Figure 1E), but anatomical location was not a statistically significant variable. The cBioPortal search revealed that 3% of *n* = 159 human OSA tissues had GPR64 genetic alterations (Figure S2). Two patients had missense mutations of unknown significance, and three patients had high GPR64 mRNA expression.

### 3.3. TOX3

TOX3 staining was diffuse, and the cytoplasmic H-scores were statistically greater than nuclear H-scores (*t*-test, *p* ≤ 0.0001; Figure 2B). The number of slides in each H-score distribution category was also significantly different (Χ^2^ = 52.99, df = 2, *p* = 0.0018). Post hoc analysis using the Fisher exact approach revealed a significant difference between nuclear and cytoplasmic low scores(*p* < 0.0032) with a Bonferroni-corrected significance level of *p* = 0.008333 (Figure 2C) [54]. In one sample (1/24; 4.17%), staining was absent, 15/24 (62.50%) had low, 7/24 (29.16%) had moderate, and 1/24 (4.17%) displayed high nuclear staining (Table 2 and Figure 2C). All tissues displayed cytoplasmic staining at varying intensities. The majority had either moderate or high staining (10/24; 41.67%, and 9/24; 37.50%, respectively), and the remaining 5/24 (20.83%) samples had low cytoplasmic staining (Figure 2C). There was a positive correlation between nuclear and cytoplasmic scores (R^2^ = 0.6269; Figure 2D). Anatomical location and sex differences for nuclear and cytoplasmic H-scores were investigated, which revealed that males had significantly higher nuclear H-scores than females (*t*-test, *p* = 0.0170; Figure 2E), but anatomical location did not exhibit statistically significant differences. The cBioPortal search revealed that 4% of *n* = 159 human OSA tissues had genetic alterations, and six patients had high TOX3 mRNA expression (Figure S2). One patient with high mRNA expression also had an amplification of TOX3.

### 3.4. MMP-12

OSA tissues (*n* = 23) displayed positive MMP-12 staining in 100% of the samples, but at varying intensities. Staining was diffuse and nuclear, and cytoplasmic H-score comparisons revealed a significant difference between the two subcellular locations; staining was weaker in the nucleus compared to the cytoplasm (*t*-test, *p* ≤ 0.0001; Figure 3B). There was also a significant difference between the number of slides in each H-score distribution category (Χ^2^ = 85.63, df = 2, *p* ≤ 0.0001). Post hoc analysis using the Fisher exact approach revealed a significant difference between nuclear and cytoplasmic low (*p* < 0.0001), moderate (*p* < 0.0001), and high scores (*p* < 0.0001) with a Bonferroni-corrected significance level of *p* = 0.008333 (Figure 3C) [54]. Staining remained low within the nucleus in the majority of cases (20/23; 86.96%), in contrast, cytoplasmic staining was low in only 5/23 samples (21.74%), but moderate in 8/23 (34.78%), and high in 10/23 (43.48%) (Figure 3C). Differences in nuclear and cytoplasmic H-scores between sexes and anatomical locations were also investigated, but there were no significant differences between these variables (*t*-tests, *p* ≥ 0.05; Supplementary Figure S1B). The cBioPortal search revealed that 7% of *n* = 159 human OSA tissues had genetic alterations, three patients had high MMP-12 mRNA expression, and seven patients had amplified MMP-12 (Figure S2).

### 3.5. FOXF1

FOXF1 staining was diffuse, and overall, cytoplasmic H-scores were significantly higher than nuclear H-scores (*t*-test, *p* ≤ 0.0001; Figure 4B). There was also a significant difference in the number of slides in each H-score distribution category (low (≤61), moderate (62–122), and high (≥123); Χ^2^ = 71.43, df = 2, *p* < 0.0002). Post hoc analysis using the Fisher exact approach revealed a significant difference between nuclear and cytoplasmic low scores (*p* < 0.0001) with a Bonferroni-corrected significance level of *p* = 0.008333 (Figure 4C) [54]. The majority of FOXF1 nuclear H-scores were classified as low (15/24; 62.50%), but staining was absent in 1/24 (4.17%) and moderate in 4/24 (16.67%) (Figure 4C). All tissues (*n* = 24) displayed positive cytoplasmic staining at varying intensities. Cytoplasmic scores were higher than observed in the nucleus, with the majority of samples classified as moderate (13/24; 54.17%), and 5/24 (20.83%) as high, and only 6/24 (25.00%) classified as low cytoplasmic staining (Figure 4C). A positive correlation was also observed between nuclear and cytoplasmic H-scores (R^2^ = 0.5504; Figure 4D). Differences in nuclear and cytoplasmic H-scores between sexes and anatomical locations were also investigated, but there were no statistically significant results between these variables (*t*-tests, *p* < 0.05; Supplementary Figure S1C). The cBioPortal search revealed that 4% of *n* = 159 human OSA tissues had genetic alterations (Figure S2). One patient had a deep deletion, one patient had amplified FOXF1, and four patients had high FOXF1 mRNA expression.

## 4. Discussion

Presently, very few potential biomarkers have been identified for either human or canine OSA [56,57,58]. Therefore, the findings in this study represent a step forward in understanding the expression of GPR64, TOX3, MMP-12, and FOXF1. High mRNA expression of the four genes of interest in this study was identified in human OSA patients via cBioPortal [55]. Hence, the findings of this study may be deemed interesting for the advancement of future therapies in human medicine as well as within veterinary medicine.

### 4.1. GPR64

*GPR64* (also known as *ADGRG2*) mRNA was expressed at a significantly higher level in canine OSA tumours compared to normal bone (log2 fold change: 3.66911, *p* = 0.0009) [29], and the present study showed positive protein expression within canine OSA, too. Mutations of human aGPCRs have been observed in several cancers and bone disorders [30,31]. Receptors GPR110 and GPR56 increased proliferation and invasion in humans and correlated with poorer OSA patient survival rates [33,34]. GPR64 is also widely expressed in various bone cancer cell lines, including Ewing sarcoma, chondrosarcoma, and primitive neuroectodermal tumours, as well as osteosarcoma in people; expression differs between cell lines and cancer types [32]. GPR64 IHC on human OSA tissues showed positive expression in 76.9% of cases, with 4/13 displaying high expression rates [32], thus corroborating the results from this present study. Normal expression of GPR64 is high within the epididymis but not present in other essential tissues [59,60]. The epididymal regions are protected from circulating antibodies by the blood–testis barrier and the blood–epididymis barrier; hence, anti-GPR64 antibodies have been shown to accumulate in sarcomas, not in healthy tissues, while also avoiding targeting the normally expressed GPR64 within the epididymis in vivo [32]. Thus indicating that GPR64 has the potential to be used as a target for antibody-mediated therapies [32].

The present study identified significantly higher GPR64 protein expression within the nucleus compared to the cytoplasm, alongside another novel finding regarding significantly higher nuclear expression within males compared to females. GPR64 expression is normally in the plasma membrane, extracellular space, and the cytosol, although there is growing evidence of GPCR nuclear signalling [61] and GPR64 nuclear expression, especially in human cancers [60]. Many class A GPCRs have been identified within the nuclear membrane, as well as some class B (which GPR64 falls within) and C proteins [62,63]. Although nuclear GPCRs also have widespread occurrence, the key physiological and/or pathophysiological conditions they are involved in are predominantly within the cardiovascular and nervous systems [64]. In Ewing sarcoma, ceramide-induced cleavage of the C-terminal intracellular domain in GPR64 allows its translocation to the nucleus [65]. It is thought that some nuclear GPCRs are able to initiate similar or identical signalling pathways usually associated with their cell surface counterparts [64]. The involvement of GPR64 within the nucleus in OSA had not previously been identified; therefore, research as to whether the full-length or truncated form of GPR64 also requires further investigation.

### 4.2. TOX3

We previously identified that TOX High Mobility Group Box Family Member 3 (*TOX3*, also known as *CAGF9* or *TNRC9*) mRNA was significantly higher in canine OSA tumours compared to normal bone (Fold change: 3.77935, *p* = 0.0002) [29]. TOX3 belongs to a subclass of transcription factors from the high mobility group (HMG)-box family [66]. The HMG-box DNA-binding domains present in TOX3 have the potential to allow unwinding of target DNA and alteration of chromatin structures for DNA-dependent cellular processes, such as transcription, replication, and strand repair [67,68,69].

TOX3 can bind to the −261 to −141 section of the ABCG2 promoter region, resulting in enhanced expression of ABCG2 in colorectal cancer [35]. ABCG2 is suggested to be involved in chemoresistance, capable of effluxing cytotoxic drugs, with known substrates including doxorubicin, methotrexate, and imatinib, all of which are used routinely in OSA therapeutic regimens [70,71,72,73]. High expression of ABCG2 in human OSA tumour tissues also correlated with significantly shorter overall patient survival times compared with normal ABCG2 expression levels [36]. Investigations into the transcriptional activity of TOX3 in OSA have not yet been undertaken. ABCG2 was not differentially expressed at the mRNA level between canine OSA and non-tumour tissues (*p* = 0.55535) [29], but it was important to note that the samples used for mRNA sequencing were treatment-naïve; therefore, resistance would not have been acquired.

The Human Protein Atlas [60] identified TOX3 to be normally localised to the nucleoplasm and/or cytosol in a variety of human tissues and cells, including within prostate and cervical cells. Additionally, in people, there was moderate nuclear expression in some cases of lung, colorectal, breast, and cervical cancer, moderate cytoplasmic staining in skin, and pancreatic cancer. Interestingly, although TOX3 RNA was expressed in 10/21 human bone cancer cell lines, protein expression was not detected in the ten samples tested [60]. The present study in canine OSA tissue revealed a significant difference between nuclear and cytoplasmic H-score distributions, displaying higher protein presence within the cytoplasm compared to the nucleus. Therefore, findings in canine OSA concurred with the usual subcellular TOX3 expression locations observed in other cell types in people, but not with protein expression in human bone cancer cell lines. There was no significant difference in the nuclear and cytoplasmic H-scores of samples retrieved from axial and appendicular locations, nor between the cytoplasmic scores of males and females. However, there was a significant difference (*p* = 0.0170) between the nuclear scores of males and females. There is limited literature on the role of TOX3 in OSA; however, a higher presence within the nucleus would suggest higher transcriptional activity of TOX3. Thus, potentially increasing the tumorigenic effects of the protein within males.

Some information is known regarding TOX3 in non-OSA cancers and its functions generally. Elevated levels of TOX3 have previously been associated with a poor prognosis in HCC patients, as well as promoting oncogenesis and neoplastic development via the MAPK and epithelial–mesenchymal transition (EMT) signalling pathways [74]. Overexpression of the gene significantly enhanced the proliferative and migratory nature of cancer cells, and knockout resulted in decreased HCC cell migratory capacity, indicating its potential involvement in metastatic progression [74].

Direct inhibition of TOX3 is not documented, although drugs such as histone deacetylases (HDAC, such as Trichostatin A, Vorinostat, and Romidepsin) have the potential to indirectly influence the transcriptional activity of TOX3 by altering histone acetylation, leading to changes in the chromatin structure, obstructing the accessibility of TOX3 to target genes [37,38]. Seiden et al. (2025) investigated Romidepsin as a potential chemotherapeutic agent for both canine and human OSA patients with metastatic disease for use in combination with current therapeutic protocols [75]. Romidepsin’s exact mechanism of action was not identified in this study; however, in vitro investigations displayed the induction of cell cycle arrest and apoptosis in 2D models, as well as inhibition of tumour growth and metastases in murine models [75].

### 4.3. MMP-12

We previously identified *MMP-12* as being significantly overexpressed in canine OSA tumours compared to non-tumour tissues (Fold change: 3.78547, *p* = 0.0001) [29]. *MMP-12* (Matrix metallopeptidase 12) is a protein-encoding gene from the peptidase M10 family, predominantly secreted by macrophages [76,77,78,79,80]. MMPs are zinc-dependent enzymes and are the major proteases in the degradation of the extracellular matrix within normal physiological processes [81]. Extracellular matrix degradation is an important process and has links to embryonic development, angiogenesis, cell repair, and tissue remodelling [81]. Abnormal degradation can occur when MMPs are differentially expressed, which can result in chronic degenerative disorders, such as arthritis, diabetes induced vascular disorders, neurodegeneration, and neoplastic progression [81].

*MMP-12* is mainly expressed in lymphoid tissue due to its involvement in immune responses; it promotes macrophage proliferation via the ERK/P38 MAPK pathway [82]. Although *MMP-12* overexpression has been observed in LUAD, colorectal cancer, and HCC [39,40,41,42], the clinical significance of MMP-12 may not prove consistent across all cancer types, as it can exert either anti-tumour or pro-tumour effects depending on the neoplasm in which the peptidase is elevated. In human OSA, MMP-12 was elevated in interleukin-13 (IL-13)–induced TAM polarisation to an M2-like phenotype, suggesting MMP-12 in OSA has pro-tumorigenic effects [43]. This work indicated that while M2 macrophages enhance the dissemination of OSA tumours to the lungs, they pose as therapeutic targets to reduce metastasis [43]. All-trans retinoic acid (ATRA) inhibited M2 polarisation of TAMs and downregulated secretion of MMP-12 from IL13-induced macrophages [43]. IL-13 and IL-14-induced M2-like macrophages, and the consequent migration of OSA cells promoted by these polarised macrophages, were suppressed by ATRA treatment [43]. There is potential for similar drug treatments to be implicated in canine OSA, given the expression of MMP-12 in the present study.

Osteosarcoma is most prevalent in large breeds and heavier individuals, with increased height in people also being a risk factor for lesion development [6]. Many studies have been conducted in human medicine investigating the correlation between height and fracture risk [6]. For example, taller women are more prone to fractures due to the assembly of wider bones with more porous and thinner cortices [83]. Later puberty may be associated with lower bone mineral density, increased osteoporosis, and fracture risk [84,85]. Obesity in both humans and canines is a major risk factor for OSA development, but also increases the risk of fracture formation, especially in patients with elevated levels of visceral adipose tissue (also contributing to lower bone mineral density, increasing the risk of fractures) [86]. The predisposed groups show similarities regarding decreased bone quality and fracture formation, and it is noteworthy to mention that MMP-12 plays a role in wound healing and tissue remodelling [87]. It may be possible that OSA develops as a result of microinjury and that MMP-12 expression, as shown in this study and our previous work [29], may form part of the bone remodelling process.

Aderamastat (FP-025) is a small molecule, highly selective MMP-12 inhibitor developed for the treatment of asthma, chronic obstructive pulmonary disease (COPD), and pulmonary fibrosis, and showed minimal adverse side effects in healthy patients (NCT03304964) [44,45]. FP-025 showed a high affinity to inhibit MMP-12 in vitro, with a 90-fold selectivity over other MMPs [44]. However, there is currently no literature regarding its use in cancer. AZD1236 is a dual MMP-9/MMP-12 inhibitor that has been investigated for its use in COPD (NCT00758459), but results regarding its efficacy require longer-term studies [46].

The results of this study showed that there were no sex or location differences in nuclear and cytoplasmic staining intensities. There was a higher protein presence within the cytoplasm across all samples, with very limited expression within the nucleus. *MMP-12* has previously been identified as a novel biomarker in other studies due to its high prevalence in many other cancers [42]. Due to the high expression of *MMP-12* in canine OSA samples compared to non-malignant counterparts (*p* = 0.0001) [29], and the work shown in the present study, there is potential for this protein to be used as a prognostic and diagnostic biomarker in this cancer type, as well as a therapeutic target. MMP-12 can be detected in blood samples; however, it is unclear as to whether elevated levels of MMP-12 within tumours are translated and are detectable within circulation [88]. Hence, this required further investigation to establish whether blood samples can also be used as a diagnostic and prognostic tool.

### 4.4. FOXF1

Forkhead box F1 (*FOXF1*) was identified as being significantly overexpressed (Fold change: 3.8936, *p* = 0.0005) in canine OSA tissues compared to non-malignant counterparts [29] and the protein was expressed in the present study. FOXF1 belongs to the forkhead box family (FOX), a subgroup of helix-turn-helix transcription factors identifiable via their distinct, highly conserved ‘fork-head’ and ‘winged-helix’ domains [89,90]. FOXF1 is also involved in the regulation of proliferation, apoptosis, inflammation, fibrosis, and EMT [91].

In canine OSA, RNA sequencing analysis revealed FOXF1 expression was elevated in tumour tissues compared to non-malignant counterparts (*p* = 0.0005) [29], and the present study showed protein expression. Similar results were also observed in human OSA cell lines and tumour samples, and patients with high FOXF1 expression in human OSA tumours also presented with lung metastasis [47]. FOXF1 could act as an independent prognostic factor in human OSA, with lower expression levels corresponding to better survival of patients [47]. siRNA-mediated knockdown of FOXF1 in human MG63 and 143B OSA cell lines suppressed neoplastic cell proliferation, migration, invasion, and tumour growth both in vivo and in vitro [47]. Knockdown of FOXF1 in these cell lines also revealed a decrease in expression, at the mRNA and protein level, of MMP-2 and MMP-9 [47]. Studies have been conducted targeting both MMP-2 and MMP-9 in osteosarcoma, which also show anti-metastatic effects [92,93]; hence, it is unclear as to whether the knocking down of FOXF1, or the decreased expression of MMP-2 and MMP-9, is what is causing these anti-tumour effects within the cell lines. In contrast, other studies suggest that FOXF1 has tumour suppressive effects in other human OSA cell lines [94]. Tamura et al. (2014) demonstrated that knockdown of FOXF1 in the Saos2 OSA cell line promoted cancer cell invasion and migration [94]. These opposing results suggest that FOXF1 functions in different ways depending on the individual tumour and that further investigation is required into the role of FOXF1 in canine OSA pathogenesis.

The importance of FOXF1 has been shown in other cancer types, making it a potential multi-cancer biomarker. In breast cancer, overexpression of FOXF1 has been shown to lead to the activation of P38 MAPK signalling and is involved in regulating SMAD2 signalling [48]. Lysyl oxidase (LOX) is an extracellular enzyme that is positively regulated by FOXF1 in breast cancer [48]. In addition, elevated levels of FOXF1 increased mammary epithelial cell invasion in vitro in a LOX-dependent manner [48]. In contrast, other studies have suggested FOXF1 has anti-tumour effects in breast cancer and suppresses cellular proliferation via the inhibition of the CDK2-RB-E2F cascade, resulting in the consequent blocking of the G1-S cell cycle transition [49].

Normal subcellular location of FOXF1 expression in humans is within the nucleus; however, the present study revealed that FOXF1 had significantly higher protein expression within the cytoplasm of canine OSA cells (Chi-squared: *p* = 0.0002). FOXF1 has also been expressed within the stroma of human lung cancer, specifically within cancer-associated fibroblasts (CAFs), with FOXF1 also being a direct target of hedgehog signalling [95,96]. There is a growing interest in CAFs as a therapeutic target for cancer due to their ability to stimulate many tumorigenic processes, such as tumour growth, invasion, angiogenesis, and metastasis [95,97]. Additionally, FOXF1 is a mesenchymal target of hedgehog signalling, and due to its association with CAFs and the induction of this signalling pathway, there is potential for this protein to contribute towards the development of novel CAF-targeted drug therapies in lung cancer, and other cancer types such as OSA [95].

Some future areas of study in relation to these potential biomarkers and OSA include analysing potential differences in relation to tumour grade, stage, T-substages I and II, invasion status, both intramedullary and extramedullary, and metastasis. Rothzerg and coauthors [98] highlighted the importance of investigating each subtype, expression profiles, and their underlying pathophysiologies, as well as developing new biomarkers to facilitate the development of novel treatments and to help detect and monitor disease. Although work is ongoing in both canine and human OSA to fully appreciate the different subtypes, understanding the potential biomarkers we have presented, and others, in different subtypes and cell types is valuable future research. Furthermore, OSA and their metastases are heterogeneous, with differing TMEs, cell types, expression levels of genes and proteins, and genomic mutations, which can lead to therapeutic challenges such as drug resistance and increased metastasis [6,99]. This further highlights the need for personalised, multi-target therapies, biomarkers in both human and canine OSA. The canine specimens in the present study have shown large ranges of proteins within the tumours, supporting their heterogeneity in terms of GPR64, TOX3, MMP-12, and FOXF1 expression. In addition, the genetic alteration rates of the four genes of interest in humans are variable. Our cBioPortal results revealed that 3–7% of *n* = 159 human OSA tissues had GPR64, TOX3, MMP-12, and/or FOXF1 genetic alterations, including missense mutations, deep deletions, high mRNA expression, and amplifications. These findings likely reflect both the heterogeneity of OSA and the differing subtypes present in the cohort, highlighting the need for personalised medicine and more advanced diagnostic and detection methods.

In order to further understand GPR64, TOX3, MMP-12, and FOXF1 in OSA, appropriate antibodies and protocols, including standardised fixation, staining, and analysis, would need to be validated prior to clinical use. Comparing against a range of non-tumour, matched tissues, specific for each antibody, could also provide insights into differential expression between not only these tissue types and subtypes but also potentially into differing anatomical locations of the bone. It would also provide essential validation for the antibodies tested, as none have previously been tested in canine tissues. Further work on protein quantification or semi-quantification using techniques such as Western blotting, mass spectrometry, and ELISA may also provide valuable validation or work as diagnostic or prognostic methodologies; however, conclusions must take into account tissue heterogeneity.

The present study investigated Rottweiler patients, but research into other breeds, and indeed other species, could be valuable in understanding the clinical applications of GPR64, TOX3, MMP-12, and FOXF1 biomarkers or therapeutic targets. Research has shown that they are expressed in a range of human cancers, and our work has shown that genetic alterations are present in 4–7% of an OSA human patient cohort. As all four proteins are expressed in a range of cancers, as such they could potentially serve as multi-cancer biomarkers. Expression could differ between species and between cancer types; therefore, individual verification for each protein, within each species and cancer, would be necessary, as with all biomarkers. In people, there is also a range of drugs targeting the proteins of interest, which provides possible routes for clinical trials in OSA patients. In dogs, we have shown GPR64, TOX3, MMP-12, and FOXF1 expression via quantitative H-scoring, described qualitative staining patterns, and investigated differences between the anatomical location of the bone tumours in both sexes. Future work to develop and validate clinical tests and understand potential expression differences between individuals, subtypes, and cell types, in different breeds, would help advance this research into pathology laboratories. Different cancer types could also be investigated in dogs, given the more extensive evidence present in human cancers of GPR64, TOX3, MMP-12, and FOXF1 expression. Given that H-scoring in veterinary medicine is not as standard as in human medicine, there is more work to be conducted in this area in general, too. Likewise, work progressing treatments in OSA, perhaps repurposing present drugs, would offer more insights into treating this complex cancer in dogs.

## 5. Conclusions

Immunohistochemical and H-scoring techniques are currently only used widely in human medicine to help determine disease diagnosis and prognosis; however, this present study highlights the relevance of the use of these ancillary techniques in veterinary medicine. This study’s results emphasise the impending use of biomarkers to aid diagnosis and be significantly relevant in certain genders, predisposing or enhancing the progression of the cancer in these animals. Deeper investigations into the overexpressed genes, *GPR64*, *TOX3*, *MMP-12*, and *FOXF1*, via the use of IHC, revealed novel findings into the cellular expression of their corresponding proteins within tumours. Analysis of these results allowed for the interpretation of potential molecular mechanisms that could be involved in canine OSA pathogenesis. Furthermore, high mRNA expression and genetic alterations of the four genes of interest in human OSA patients indicated high potential for translation into human medicine. A deeper understanding of both the protein interactions and the genetics of OSA, and their mechanisms, is needed in order to discover and utilise novel diagnostic, prognostic, and treatment opportunities. Additionally, these novel findings within a veterinary context may have translational abilities within human medicine, showcasing the ‘One Health’ applicability of research.

## Figures and Tables

**Table 1:** H-scores for GPR64, TOX3, MMP-12, and FOXF1.

Protein (no. of Cases)	Staining Distribution (Diffuse/Multifocal/Focal)	Cellular Location	H-Score	
			Mean ± SEM (2 s.f)	Range (min–max)
GPR64 (*n* = 20)	Diffuse	Nuclear	113.38 ± 9.58	5–191
		Cytoplasmic	89.10 ± 7.61	14–163
TOX3 (*n* = 24)	Diffuse	Nuclear	33.16 ± 6.25	0–104
		Cytoplasmic	77.83 ± 7.61	5–144
MMP-12 (*n* = 23)	Diffuse	Nuclear	24.38 ± 6.07	1–124
		Cytoplasmic	78.38 ± 7.66	9–142
FOXF1 (*n* = 24)	Diffuse	Nuclear	26.17 ± 6.06	0–108
		Cytoplasmic	91.08 ± 9.38	6–190

Mean H-scores for nuclear and cytoplasmic staining for each protein, as well as the standard error of the mean (SEM), and the H-score ranges for each protein (nuclear and cytoplasmic). s.f = significant figures.

**Table 2:** Subcellular staining H-scores (nuclear and cytoplasmic) for GPR64, TOX3, MMP-12, and FOXF1.

Nuclear	**Cytoplasmic**				
		**Absent**	**Low**	**Moderate**	**High**
	[GPR64, *n* = 20]				
	Absent	-	-	-	-
	Low	-	2 (10.00%)	-	-
	Moderate	-	1 (5.00%)	7 (35.00%)	-
	High	-	1 (5.00%)	8 (40.00%)	1 (5.00%)
	[TOX3, *n* = 24]				
	Absent	-	1 (4.17%)	-	-
	Low	-	4 (16.67%)	8 (33.33%)	3 (12.50%)
	Moderate	-	-	2 (8.33%)	5 (20.83%)
	High	-	-	-	1 (1.47%)
	[MMP-12, *n* = 23]				
	Absent	-	-	-	-
	Low	-	5 (21.74%)	7 (30.43%)	8 (34.78%)
	Moderate	-	-	1 (4.35%)	-
	High	-	-	-	2 (8.70%)
	[FOXF1, *n* = 24]				
	Absent	-	1 (4.17%)	-	-
	Low	-	5 (20.83%)	12 (50.00%)	2 (8.33%)
	Moderate	-	-	1 (4.17%)	3 (12.50%)
	High	-	-	-	-

The percentage of specimens in each distribution category (absent, low, moderate, or high) for each protein of interest’s mean nuclear and cytoplasmic H-scores (*GPR64*, *TOX3*, *MMP-12*, and *FOXF1*).

## Data Availability

Anonymized data is available upon reasonable request from the corresponding authors.

## Supplementary Materials

The following supporting information can be downloaded at: https://www.mdpi.com/article/10.3390/cancers18020262/s1, Table S1: Age, neutering status, and location of OSA for each specimen (*n* = 25). Figure S1: Sex and ages of the canine specimens, and MMP-12 and FOXF1 immunostaining nuclear and cytoplasmic H-score, gender, and anatomical location. Figure S2: cBioPortal search for genetic alterations in human OSA tissues TARGET GDC, 2025 cohort (*n* = 159). Genetic alterations in human OSA tissues (*n* = 159, from *n* = 153 patients) were identified for the four genes of interest in canine OSA (GPR64 (ADGRG2), TOX3, MMP-12, and FOXF1). Mutation data identified from whole-exome sequencing. Putative copy-number alterations from GISTIC 2.0. Values represent several copies of the chromosomal region compared to the diploid number of cells: −2 = homozygous deletion; −1 = hemizygous deletion; 0 = neutral/no change; 1 = gain; 2 = high level amplification. mRNA expression from capture (RNA seq TPM) zscores, threshold zscore ± 2.0.

## Abbreviations

The following abbreviations are used in this manuscript:

ABCG2	ATP Binding Cassette Subfamily G Member 2
ATRA	All trans retinoic acid
BMP	Bone Morphogenetic Protein
CAF	Cancer-Associated Fibroblast
COPD	Chronic obstructive pulmonary disease
EMT	Epithelial–mesenchymal transition
ER	Oestrogen receptor
FFPE	Formalin-fixed paraffin-embedded
FGF4	Fibroblast Growth Factor 4
FOXF1	Forkhead Box F1
FP-025	Aderamastat
GPCR	G protein-coupled receptor
GPR64	Adhesion G Protein-Coupled Receptor G2/ADGRG2
HCC	Hepatocellular Carcinoma
HDAC	Histone deacetylases
HER2	Human epidermal growth factor receptor 2
HMG-(box)	High Mobility Group- (box)
IHC	Immunohistochemistry
LOX	Lysyl oxidase
LUAD	Lung Adenocarcinoma
MAPK	Mitogen-activated protein kinase
MMP-12	Matrix Metalloproteinase-12
OR	Odds Ratio
OSA	Osteosarcoma
PR	Progesterone receptor
SEM	Standard error mean
SMOC	SPARC-related modular calcium-binding protein
TAM	Tumour-associated macrophage
TOX3	TOX High Mobility Group Box Family Member 3
TPM	Transcripts per million
