# Supplementary material for: Identification and Characterisation of Canine Osteosarcoma Biomarkers and Therapeutic Targets
Source: Cancers (Basel). 2026 Jan 14;18(2):262. doi: 10.3390/cancers18020262 (PMC12838725; doi:10.3390/cancers18020262)
Supplement: Supplementary file 1 [file cancers-18-00262-s001.zip › cancers-4053512-supplementary.pdf]

Table S1: Age, neutering status and location of OSA for each specimen (n=25).

Number	Age (Yrs)	Neutering Status	Location of Lesion
1	6.16	Mn	Temporo-mandibular joint
2	9	Fn	Gum
3	7.5	Fn	Mesenteric lymph node
4	9.75	Fn	Humerus
5	4.5	Mn	Left ilium
6	10	F (ns)	Scapula
7	10.75	Fn	Right mandible
8	12	Fn	Proximal humerus
9	9	M (ns)	Right tibia
10	12	Fn	Ulnar
11	5.83	M (ns)	Humerus
12	-	Fn	Left stifle
13	6.75	M	Muzzle
14	7.66	Fn	Left hip
15	-	M (ns)	Left hind (proximal to left stifle)
16	6.92	Fn	Right humerus
17	10	Fn	Stifle
18	9	Fn	Proximal humerus
19	7	M (ns)	Right stifle
20	10	-	Right fore
21	8	M (ns)	Oral mass
22	10.75	Fn	Stifle
23	4.83	F	Right distal femur
24	6.5	Mn	Distal tibia
25	6.66	F (ns)	Left proximal humerus

All tissue samples were formalin fixed, paraffin embedded (FFPE) blocks. Information regarding the age, neutering status, and location of each lesion is within the table. Neutering status abbreviations: F=female not neutered, Fn=female neutered, F (ns)= female neutering status not specified, M=male not neutered, Mn=male neutered, M (ns)=male neutering status not specified., n=neutered, ns=not specified.

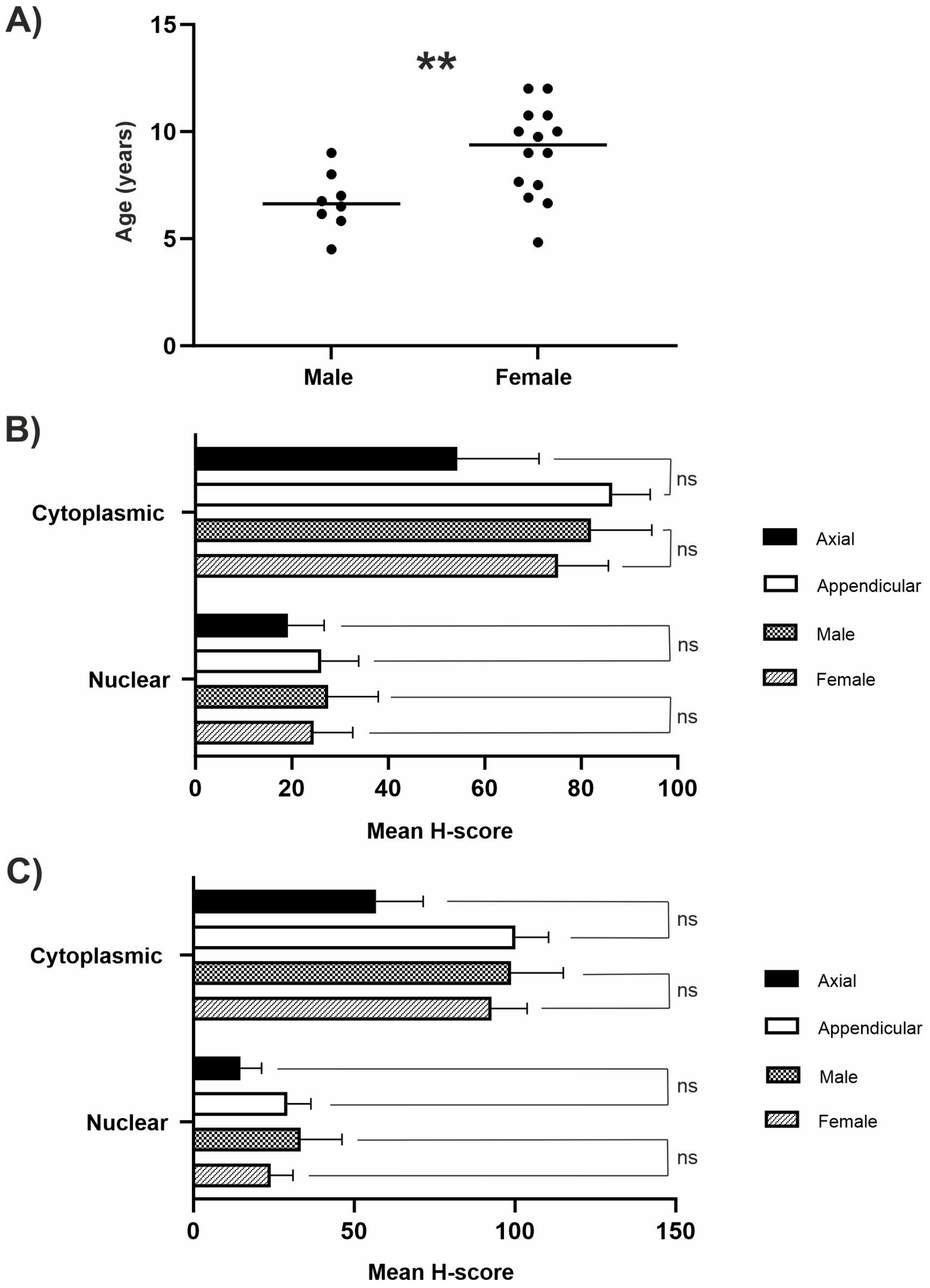

Figure S1: Sex and ages of the canine specimens, and MMP-12 and FOXF1 immunostaining nuclear and cytoplasmic H-score gender and anatomical location. (A) Male and female sample dog ages, n=22 (n=8 males, n=14 females, n=3 age or gender unknown and were not plotted within the corresponding graphs). The horizontal line indicates the mean age for each gender (male: 6.72 years and female: 9.06 years, 2 s.f.). (B) MMP12: T-tests comparing MMP-12 positive nuclear and cytoplasmic H-scores of tissues from male (n=8) versus female (n=14) dogs as well as neoplasms excised from axial (n=6) versus appendicular (n=17) locations. (C) FOXF1: T-tests comparing FOXF1 positive nuclear and cytoplasmic H-scores of tissues from male (n=8) versus female (n=15) dogs as well as neoplasms excised from axial (n=5) versus appendicular (n=19) locations. T-test: p≥0.05 (ns), p<0.05 (*), p<0.01 (**), p<0.001 (***), p<0.0001 (****).

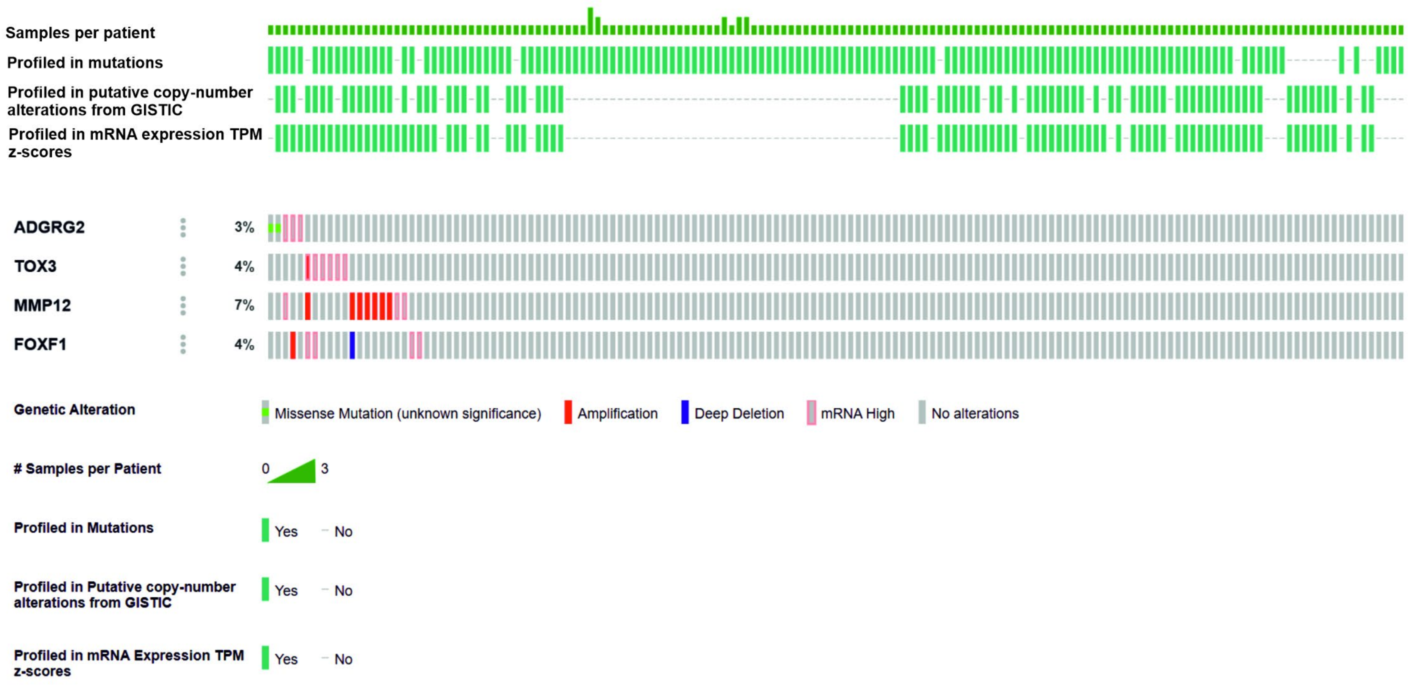

Figure S2: cBioPortal search for genetic alterations in human OSA tissues TARGET GDC, 2025 cohort (n=159). Genetic alterations in human OSA tissues (n=159, from n=153 patients) identified for the four genes of interest in canine OSA (GPR64 (ADGRG2), TOX3, MMP-12, and FOXF1). Mutation data identified from whole exome sequencing. Putative copy-number alterations from GISTIC 2.0. Values represent are number of copies of the chromosomal region compared to the diploid number of cells: -2 = homozygous deletion; -1 = hemizygous deletion; 0 = neutral/no change; 1 = gain; 2 = high level amplification. mRNA expression from capture (RNA seq TPM) zscores, threshold zscore ± 2.0.
